# A review of acquired blepharoptosis: prevalence, diagnosis, and current treatment options

**DOI:** 10.1038/s41433-021-01547-5

**Published:** 2021-04-29

**Authors:** Jason Bacharach, Wendy W. Lee, Andrew R. Harrison, Thomas F. Freddo

**Affiliations:** 1 North Bay Eye Associates, Petaluma, CA USA; 2 Bascom Palmer Eye Institute, University of Miami Miller School of Medicine, Miami, FL USA; 3 Department of Ophthalmology and Visual Neurosciences, Department of Otolaryngology, University of Minnesota, Minneapolis, MN USA; 4 Massachusetts College of Pharmacy and Health Sciences, Worcester, MA USA

**Keywords:** Eyelid diseases, Surgery, Drug therapy

## Abstract

Blepharoptosis (ptosis) is among the most common disorders of the upper eyelid encountered in both optometric and ophthalmic practice. The unilateral or bilateral drooping of the upper eyelid that characterises ptosis can affect appearance and impair visual function, both of which can negatively impact quality of life. While there are several known forms of congenital ptosis, acquired ptosis (appearing later in life, due to a variety of causes) is the predominant form of the condition. This review summarises the prevalence, causes, identification, differential diagnosis, and treatment of acquired ptosis. Particular attention is paid to the differential diagnosis of acquired ptosis and emerging treatment options, including surgical and pharmacologic approaches.

## 摘要

上睑下垂是视光学和眼科临床中最常见的上睑疾病之一。以上睑下垂为特征的单侧或双侧上睑下垂会影响外观和视觉功能, 这两种情况都会对生活质量产生负面影响。目前已知有几种先天性的上睑下垂, 但获得性上睑下垂 (由各种原因所导致并出现在其后的生活中) 是该病的主要形式。本文就获得性上睑下垂的发病率、病因、鉴别诊断及治疗作一综述。并特别研究获得性上睑下垂的鉴别诊断以及新的治疗方法, 包括手术和药理治疗途径。

## Literature search notes

Literature cited in this review was identified via a broad search of the PUBMED online database for English-language, peer-reviewed publications including search terms such as “ptosis,” “epidemiology,” “etiology,” “eyelid,” “surgical,” “pharmacologic,” “Müller’s muscle,” “adrenergic,” “visual field,” and “quality of life.” Relevant primary and review articles were reviewed and cited when providing unique primary data or a current summary of fundamental concepts. Also included, when relevant, were primary or review articles not identified via PUBMED, but cited in publications retrieved via this literature search.

## Acquired ptosis overview, prevalence, and impacts

Blepharoptosis, more commonly known as “ptosis,” is an abnormal drooping of the upper eyelid with the eye in primary gaze. This drooping can affect one or both eyes, and based on time of appearance, it is broadly classified as either congenital (present at or shortly following birth) or acquired (appearing later in life). Ptosis is broadly recognised as being among the most common disorders of the eyelid encountered in the clinic, however data from large population-based studies are limited. Estimates of ptosis prevalence are largely based on data from region-specific studies, which report rates between 4.7 and 13.5% in adult populations and support the widespread nature of the condition [1–3]. Furthermore, these studies consistently reveal that, within adult populations, the incidence of ptosis increases with age (Table 1 and Acquired ptosis risk factors). Reports of ptosis incidence in surgical populations are consistent with those in broader patient populations. In a study evaluating a cohort of 623 patients referred for surgery in an oculoplastics department in Singapore, ptosis was the most common condition, occurring in 11.7% of patients [4].

**Table 1:** Studies reporting on the prevalence of ptosis in the general adult population.

Study	Location	Subjects evaluated	Ptosis prevalence				Other associated factors
			Overall	By sex	By age	Eye(s) affected	
Forman et al. 1995 [1]	United Kingdom	*N* = 400 adults ≥50 years old (166 M; 234 F)	11.5%	• M: 9.0% • F: 13.2%	• 50–59 years old: 2.4% • 60–69 years old: 8.9% • 70–79 years old: 12.5% • ≥80 years old: 42.9%	• 57% of cases bilateral	• n/a
Hashemi et al. 2016 [2]	Iran	*N* = 4737 adults 44-69 years old (1946 M; 2791 F)	4.7%	• M: 4.0% • F: 5.2%	• 45–49 years old: 3.1% • 50–54 years old: 3.7% • 55–59 years old: 4.7% • 60–64 years old: 7.1% • 65–69 years old: 5.8%	• 27.7% of cases bilateral	• Hypertension, diabetes
Kim et al. 2017 [3]	South Korea	*N* = 17,286 adults ≥40 years old (48.8% M; 51.2% F)	13.5%	• 50.1% of patients with ptosis were F	• 40–49 years old: 5.4% • 50–59 years old: 11.6% • 60–69 years old: 19.8% • ≥70 years old: 32.8%	• Not specified	• Hypertension. diabetes, higher BMI, history of CV disease, hyperopia, strabismus, cataract

*BMI* body mass index, *CV* cardiovascular.

Drooping of the upper eyelid due to ptosis can lead to the condition’s characteristic ‘sleepy’ appearance, as well as asymmetry, in both unilateral and bilateral cases [5, 6]. Studies reveal that this can have important impacts on patient well-being, including reduced independence and increased appearance-related anxiety and depression [7, 8]. In a study in the United Kingdom, adults referred for ptosis surgery were assessed prior to surgery using validated questionnaires addressing psychosocial factors, including appearance-related distress (the Derriford Appearance Scale (DAS 24)), anxiety and depression (the Hospital Anxiety and Depression Scale (HADS)), fearful or worrying conditions related to the perceived opinions of others (the Fear of Negative Evaluation (FNE) Scale), and self-evaluation of appearance (the Centre for Appearance Research Valence (CARVAL) scale). Patients reported levels of appearance-related distress, anxiety, and depression that were higher than typical norms in the general population and similar to levels previously reported in patients with other appearance-altering ophthalmic conditions, such as strabismus [8]. The analysis also identified significant gender differences with respect to DAS 24, HADS, FNE, and CARVAL scores, with female patients reporting higher mean scores than males [8].

From a functional perspective, obstruction of the pupil as a result of ptosis can lead to deficits in the superior visual field, detectable via visual field testing and evident even in mild cases [9–11]. An evaluation of the superior visual field using static perimetry testing (Humphrey Visual Field (HVF) Test) in subjects at baseline and after induction of mild or moderate ptosis using eyelid weights found that even mild ptosis was associated with significant depression of all test points along the superior hemifield, and that this worsened in the moderate ptosis condition [11]. Among more recent studies in patients with ptosis, a study validating a novel static perimetry test (the Leicester Peripheral Field Test (LPFT)) revealed that 84 of 85 ptotic eyes had a visual field deficit [10]. Visual field testing methods are described in detail the section titled Acquired ptosis identification and differential diagnosis.

The effect of ptosis goes beyond diminished performance on visual field tests. Visual field loss is associated with decreases in health-related quality of life (HRQoL) measures [7], indicating meaningful impacts on patients’ daily lives. In the Los Angeles Latino Eye Study (LALES), more than 5200 subjects underwent ophthalmic examination and visual field testing. Data from this population revealed that greater visual field loss, measured using the HVF Test, correlated with worse scores on two validated tools to assess HRQoL—the Medical Outcomes Study 12-item Short-Form Health Survey (SF-12) and the National Eye Institute Visual Function Questionnaire (NEI-VFQ-25). While bilateral moderate/severe visual field loss was associated with the greatest negative effect on HRQoL measures, decreases in HRQoL were also evident in participants with mild unilateral visual field loss [7]. The reduction in HRQoL was found to be, at least in part, due to the reduction in independence (greater difficulty driving and performing regular tasks) that arises due to visual field deficits [7]. Studies also show that improvements in subjective and objective visual performance following intervention are associated with improved HRQoL-related measures [12, 13]. In a study of 50 patients who underwent ptosis surgery, patients showed significant improvement versus pre-surgery assessment, with respect to a range of vision-related activities and symptoms, including the ability to perform fine manual work, hang or reach objects above eye level, watch television, and read [12]. Similarly, in a study of 100 patients with unilateral or bilateral ptosis that used the same questionnaire, improvement in the superior visual field following surgery was associated with a greater functional index, and patients had significant improvement with respect to activities including performing their occupation, playing sports, and walking without assistance [13].

## The upper eyelid and causes of acquired ptosis

**Fig. 1:**
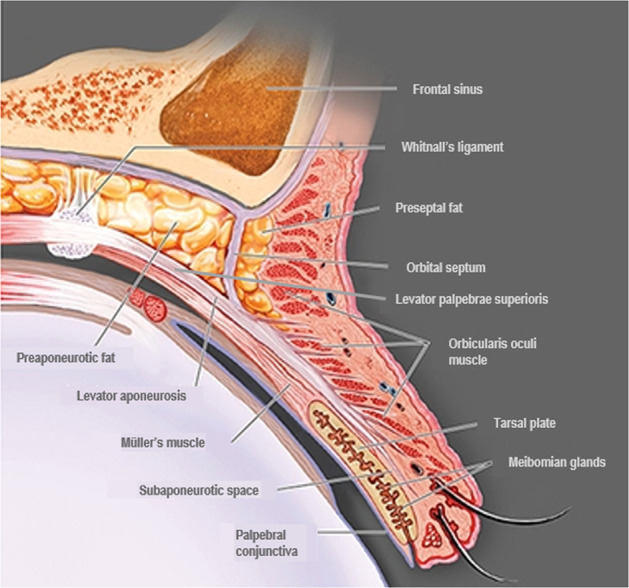
Anatomy of the upper eyelid. Adapted from Freddo and Chaum, 2017 [14]. The striated levator palpebrae superioris is innervated by the oculomotor nerve (cranial nerve III) and inserts, through its aponeurosis, on the anterior surface of the superior tarsal plate. Except in the eyelids of Asian individuals, the aponeurosis extends fibres through the orbicularis oculi muscle to reach the skin of the upper eyelid. The smooth Müller’s muscle arises from the underside of the levator and inserts on the superior tarsal plate. It is innervated by sympathetic fibres from the superior cervical ganglion [5, 14–16].

Elevation of the upper eyelid is largely provided by two muscles—the levator palpebrae superioris (levator) and the superior tarsal (Müller’s) muscle (Fig. 1). The levator is a voluntary (striated) muscle that originates from the lesser wing of the sphenoid bone at the orbital apex and inserts, through its aponeurosis, onto the anterior surface of the superior tarsal plate. It also has attachments to the skin of the upper eyelid, which contribute to the formation of the lid crease. This insertion is absent or poorly formed in some Asian individuals. The levator is innervated by the superior division of the oculomotor nerve (cranial nerve III), and its contraction provides the majority (~80%) of upper eyelid elevation [5, 14–16]. Müller’s muscle arises from the underside of the levator, at the level of the distal aponeurosis, and inserts onto the superior tarsal plate [5, 14, 15]. In contrast to the striated levator muscle, Müller’s muscle—like its analogue in the lower eyelid, the inferior tarsal muscle—is an involuntary (smooth) muscle. With contraction, Müller’s muscle helps to sustain upper eyelid elevation provided by the levator, while also supplying 1–2 mm of additional lift [5, 14]. Similarly, in the lower eyelid, the inferior tarsal muscle assists in lowering the lid during downward gaze, though there is no striated muscle analogous to the levator. Both Müller’s muscle and the analogous inferior tarsal muscle receive sympathetic innervation from nerve fibres originating in the superior cervical ganglion [5, 14, 15]. A study of adrenergic receptor expression in Müller’s muscle revealed a predominance of the α_2A_ subtype, and lower expression of the α_1_ and β_1_ subtypes [17]. Further examination of receptor subtype expression in Müller’s muscle has also demonstrated expression of the α_1D_, α_2C_, and β_2_ subtypes in patients with ptosis [18, 19]. In contrast to Müller’s muscle, the levator predominantly expresses the β_1_-adrenergic receptor subtype, with only trace expression of the α_1_, α_2_, and β_2_ subtypes [17].

The frontalis muscle, which inserts at the level of the eyebrows, is innervated by the facial nerve (cranial nerve VII) and its contraction raises the brow, with no direct effect on upper eyelid elevation. In patients with ptosis, however, compensatory raising of the brow via the frontalis muscle can indirectly provide slight elevation of the eyelid as well [15].

Broadly, ptosis is classified based on time of onset. Congenital ptosis (present at birth) typically has a unilateral presentation and is most often a result of developmental myopathy of the levator muscle that affects the levator’s ability to contract and raise the upper eyelid [20–22]. Neurogenic forms of congenital ptosis can be caused by cranial nerve III abnormalities or insufficient sympathetic innervation of Müller’s muscle. Furthermore, several craniofacial syndromes or cranial dysinnervation disorders can also underlie congenital ptosis, including Marcus Gunn jaw-winking syndrome or blepharophimosis [22, 23].

Acquired ptosis, the predominant form of ptosis (Table 2), can be classified by aetiology, with cases typically defined as having an aponeurotic, myogenic, neurogenic, mechanical, or traumatic origin. Aponeurotic ptosis, the most common acquired form of the condition [24], is caused by stretching, dehiscence, or detachment of the levator aponeurosis from its insertion on the tarsus, and is typically associated with aging [5, 16, 24]. Myogenic ptosis is caused by primary or secondary myopathy of the levator muscle, due for example to chronic progressive external ophthalmoplegia (CPEO), oculopharyngeal muscular dystrophy (OPMD), or myotonic dystrophy [5, 16, 22]. Neurogenic ptosis is relatively rare and is typically caused by dysfunction or damage to the oculomotor nerve or to sympathetic nerves innervating the eyelids, or by central mechanisms [5, 16, 22]. Among patients with neurogenic ptosis, the most common underlying causes are oculomotor nerve (3^rd^ cranial nerve) palsy (35.7%), myasthenia gravis (28.6%), aberrant regeneration (14.3%), and Horner’s syndrome (7.1%) [24]. Common causes of mechanical ptosis include benign or malignant neoplasms of the eyelid, such as haemangioma, chalazion, neurofibroma, or dermoid cysts, which create excess weight that cannot be raised by the upper eyelid retractor muscles [5, 22]. Finally, acquired ptosis can arise due to trauma to the eyelid retractor muscles, aponeurosis, or neural inputs to the eyelid. Thus, traumatic ptosis can be myogenic, aponeurotic, or neurogenic in nature [22].

**Table 2:** Summary of common types of acquired ptosis, causes, and ptosis risk factors.

Types of acquired ptosis	
Aponeurotic (involutional)	• Caused by stretching, dehiscence, or detachment of the levator aponeurosis, and is typically associated with aging [5, 16, 24] • Typically presents with reduced MRD-1, high upper eyelid crease, near normal levator function, and decreased PFD [24]
Myogenic	• Caused by primary or secondary myopathy of the levator muscle (e.g., due to OPMD, myotonic dystrophy, or CPEO) [5, 16, 22] • Can present with a weak/absent upper eyelid crease, poor levator function, and eyelid lag on downgaze [24]
Neurogenic	• Relatively rare type of ptosis, caused by CNS abnormality or underlying neurological condition affecting the oculomotor or sympathetic nerves [5, 16, 22] • Can have a range of presentations, depending on underlying cause (e.g., Horner’s syndrome presents as unilateral ptosis with ipsilateral pupil constriction and facial anhidrosis) [5, 16, 24]
Mechanical	• Caused by excess weight on the upper eyelid, usually due to benign or malignant neoplasm (e.g., haemangioma, chalazion, neurofibroma, dermoid cyst) [5, 22] • Can also be a ‘pseudoptosis’ (e.g., dermatochalasis, in which levator function is not impaired) [5, 16, 22]
Traumatic	• Caused by trauma to the eyelid retractor muscles, aponeurosis, or neural inputs to the eyelid [22] • Can be myogenic, aponeurotic, or neurogenic in nature [22]
Common environmental risk factors for acquired ptosis	
Age	• In adult populations, prevalence of ptosis increases with age [1–3] • Age-related aponeurotic ptosis the most common form of ptosis in older adult patients referred for ptosis surgery [24, 25]
Contact lens wear	• Long-term wear of hard or soft contact lenses associated with increased ptosis risk [25–29]
Ocular surgery	• Transient or persistent ptosis associated with a range of procedures, including glaucoma, cornea, strabismus, and cataract surgery [30, 31, 33, 34] • Risk can be dependent upon surgical technique used [31, 33, 34]
Periocular neurotoxin injection	• Botulinum toxin injections associated with transient upper eyelid ptosis [36–38]

*CNS* central nervous system, *CPEO* chronic progressive external ophthalmoplegia, *MRD-1* Marginal Reflex Distance 1, *OPMD* oculopharyngeal muscular dystrophy, *PFD* palpebral fissure distance.

Pseudoptosis does not involve pathology of the upper eyelid retractor muscles or aponeurosis, and can be due to mechanical, neurogenic, or anatomical causes. Mechanical causes include dermatochalasis (excessive upper eyelid skin that overhangs the lid margin), brow ptosis (drooping of the eyebrow), and floppy eyelid syndrome (easy eversion of the upper eyelid due to excessive lid laxity). Neurogenic causes include benign essential blepharospasm and hemifacial spasm (unilateral spasm of the upper and lower eyelids). Anatomical causes include microphthalmos (decreased size or volume of the globe) or superior sulcus deformity (deepening of the superior sulcus) [5, 16, 22]. Diagnostic differentiation of acquired ptosis is discussed in the section titled Acquired ptosis identification and differential diagnosis.

## Acquired ptosis risk factors

Studies of adult populations consistently reveal age as a significant risk factor for the development of acquired ptosis, with reported prevalence exceeding 20% among patients aged 70 years and older (summarised in the section titled Acquired ptosis overview, prevalence, and impacts and in Table 1) [1–3]. In a 1995 study of 400 individuals ≥50 years old in the United Kingdom, 11.5% were determined to have ptosis, with the relative frequency increasing from 2.4% among individuals aged 50–59 years to 8.9% among individuals aged 60–69 years old and 20.8% among individuals aged ≥70 years [1]. A more recent study of >4700 Iranian patients 45 to 69 years old reported an incidence of 4.7%, with the lowest prevalence (3.1%) among patients aged 45–49 years and the highest prevalence (7.1%) among patients aged 60–64 years [2]. Another study, of 17,296 patients ≥40 years old in Korea, reported an overall prevalence of ptosis of 13.4%, with the lowest prevalence (5.4%) among patients 40–49 years old and the highest prevalence (32.8%) among patients ≥70 years old [3]. These two studies also reported higher rates of ptosis in patients with diabetes and hypertension [2, 3]. Furthermore, the Korean study found an association between higher body mass index (BMI), as well as a history of cardiovascular disease, and the presence of ptosis [3]. Individuals with ptosis in this study were also found to be more likely to have hyperopia, strabismus, and cataract, in comparison to individuals without ptosis [3].

In an analysis of 251 patients referred for ptosis surgery to an ophthalmic surgery centre in Singapore, aponeurotic ptosis was the most common form of ptosis observed (60.2%). In this study, the median age among patients with aponeurotic ptosis was 62 years. The other most common forms observed in the study were traumatic (11.2%), congenital (10.4%), mechanical (8.8%), neurogenic (5.6%), and myogenic (4.0%) ptosis [24]. Similarly, an evaluation of patients presenting at an oculoplastic surgery practice in Australia revealed that involutional (aponeurotic) ptosis was the most common form among patients over 50 years of age, accounting for 17% of cases among patients aged 51–60 years, 34% of cases among patients aged 61–70 years, and 31% of cases among patients aged 71–80 years [25].

Contact lens wear, which involves repeated manipulation of the eyelid, and therefore the potential risk of microtrauma to the levator aponeurosis, has also been associated with the development of acquired ptosis, with studies linking both hard and soft contact lens wear to increased incidence (Table 2) [25–29]. A retrospective analysis of 15 patients with ptosis attributable to contact lens wear revealed that all were hard lens wearers and 13 of 15 had been wearing their lenses for >17 years. Furthermore, in 11 of the 15 patients, thinning or dehiscence of the levator aponeurosis was observed during surgery [25]. Along similar lines, an age-matched case-control study of female patients in Japan found that hard contact lens wear significantly increased the risk of ptosis versus non-wear (odds ratio 19.9 (6.32–62.9)) [28]. A retrospective analysis of 35 patients aged 18–50 years old presenting with ptosis in a hospital ophthalmology department found that 29 of the 35 patients had a history of either hard or soft contact wear (mean wear time 17.6 and 9 years, respectively) [26]. A broad analysis of environmental factors contributing to ptosis in 286 sets of adult twins (range: 18–82 years old) found a significant association between both hard and soft contact lens wear and ptosis, but no association with respect to other environmental factors evaluated, including BMI, smoking status, alcohol consumption, hours of sleep per night, or sun exposure [29]. Consistent with these individual studies, a systematic literature review found significant risk associated with both hard (odds ratio 17.38 (3.71–81.29)) and soft (odds ratio 8.12 (2.68–24.87)) contact lens wear [27].

Another known cause of ptosis is ocular surgery. A systematic literature review reported an 11.4% incidence of ptosis following ocular surgery, with the highest rate (13.4%) occurring among patients who underwent glaucoma surgery, followed by corneal (10.3%), strabismus (10.0%), cataract (9.4%), and mixed (6.5%) surgeries [30]. Postsurgical ptosis incidence also depends on surgical technique [28]. Reported rates of ptosis range from 1 to 44.4% and 0 to 12.9% among patients following extracapsular and phacoemulsification cataract surgery, respectively. Similarly, incidence after glaucoma surgery (7–19%) and vitreoretinal procedures (9.7–17%) appears to depend on the surgical technique used [31]. In glaucoma surgery, reported ptosis incidence is higher in trabeculectomy with mitomycin C (19% incidence) than when mitomycin is not used (12%) [31]. In vitreoretinal surgery, reported ptosis incidence with intravitreal steroid injection and intravitreal anti-VEGF injection with sub-Tenon’s steroid injection are reported to be 11% and 17%, respectively [31].

Ptosis following ocular surgery can be transient or persistent. Factors suspected of causing transient postsurgical ptosis include the occurrence of postsurgical oedema, haematoma, foreign body reaction, and use of neuromuscular blockade, while proposed mechanistic causes of more persistent postsurgical ptosis include the use of mitomycin C in glaucoma surgery, direct trauma to the tarsal plate, bridle suture use (with higher incidence occurring with a closed approach), and rigid eyelid speculum use, which can lead to levator aponeurosis dehiscence or detachment from the tarsal plate [31–35].

Similarly, transient ptosis has been reported as an adverse event following periocular neurotoxin injection [36–38]. A broad systematic literature review evaluated clinical safety data related to the use of botulinum toxin A for facial aesthetic treatment in >8700 total patients. Brow ptosis (3.1% incidence) was the most commonly reported adverse event in the upper face, followed by eye sensory disorders (3.0%), and eyelid ptosis (2.5%), with all events being transient and resolving spontaneously [36]. More recently, case series have described potential approaches to treating transient ptosis resulting from botulinum toxin injection, with, most notably, topical application of the adrenergic agent apraclonidine providing measurable upper eyelid elevation in some patients [37, 38].

Ptosis can also be secondary to a range of underlying neurological or muscular conditions, including 3^rd^ cranial nerve palsy, CPEO, oculopharyngeal muscular dystrophy, Horner’s syndrome, and myasthenia gravis [5, 16]. These conditions can range in severity and require different interventions than cases of primary ptosis due exclusively to upper eyelid retractor muscle or aponeurosis defects. These underlying conditions can also be emergent and potentially life-threatening, and therefore require rapid intervention. Ptosis identification and differential diagnosis are summarised in detail in Acquired ptosis identification and differential diagnosis, below.

## Acquired ptosis identification and differential diagnosis

Accurately identifying ptosis, as well as its underlying aetiology and severity, is essential to successful management. Thorough clinical examination and differential diagnosis is also needed in order to rule out similar conditions or most importantly, diagnose any serious underlying cause requiring more immediate medical intervention (Table 3).

**Table 3:** Diagnostic testing approaches for acquired ptosis.

Ptosis identification	
Patient history and evaluation for serious underlying conditions	• Evaluate timing of onset • If onset suggests serious underlying condition (e.g., Horner’s syndrome, 3^rd^ cranial nerve palsy, myasthenia gravis, CPEO), examine for characteristic ophthalmic or non-ophthalmic signs and perform appropriate ancillary testing (e.g., phenylephrine or apraclonidine test for Horner’s syndrome, rest test/ice test, anti-acetylcholine antibody testing for myasthenia gravis) [5, 15, 16, 40] • Evaluate periocular skin and soft tissues to identify/exclude mechanical cause or tumour [5, 16, 22]
Exclusion of pseudoptosis	• If dermatochalasis or brow ptosis present, examine upper eyelid while raising excess skin [16] • Examine the globe for dystopias (enophthalmos, hyper/hypoglobus) or asymmetry (phthisis bulbi, microphthalmia) [16] • If contralateral retraction is suspected, examine for thyroid eye disease [16]
Upper eyelid evaluation	
MRD-1	• Distance from the central pupillary light reflex to the central margin of the upper eyelid; provides a measure of ptosis severity [5, 16, 44]
Eyelid crease height	• Distance from the upper eyelid crease to the eyelid margin; increased eyelid crease height can indicate dysfunction of the levator aponeurosis [44]
Palpebral fissure height	• Distance between the upper and lower eyelid margins with the eye in primary gaze; decreased palpebral fissure height can indicate disinsertion of the levator aponeurosis from the tarsal plate [16, 44]
Levator function (Berke’s method)	• Upper eyelid excursion upon shift from downgaze to upgaze, with the frontalis muscle excluded; decreased excursion indicates greater degree of levator functional impairment (0-4 mm lid elevation = poor; 5-11 mm = fair; 12-14 mm = good; >15 mm = normal) [44]
Müller’s muscle function	• Assessed by instilling phenylephrine and measuring upper eyelid lift; more lift indicates better Müller’s muscle function [16, 44, 45, 47]
Visual field testing	
Goldmann Visual Field Test	• Observer-dependent, kinetic perimetry test, using Goldmann bowl; visual field mapped manually by the examiner [50]
Humphrey Visual Field Test	• Automated, static perimetry test using Humphrey Visual Field Analyser; 24° superior visual field tested, using a 54-point grid (24-2 setting) [10, 50]
Leicester Peripheral Field Test (LPFT)	• Modified Humphrey Visual Field Test; automated, operator-independent, static perimetry test evaluating a 48° superior field, using a 35-point grid (14-point grid in inferior field used as reference) [10] • Shifted centre of fixation allows for more natural eyelid positioning and reduction of compensatory behaviours

*CPEO* chronic progressive external ophthalmoplegia, *MRD-1* marginal reflex distance 1.

The initial diagnostic step is a review of patient history to understand timing of ptosis onset, as a sudden appearance may signal serious underlying pathology. If patient history suggests that ptosis may be secondary to a more serious condition, subsequent evaluation can be conducted based on the observable clinical signs. The serious neurological or muscular conditions most commonly encountered in clinical practice include Horner’s syndrome, 3^rd^ cranial (oculomotor) nerve palsy, myasthenia gravis, and CPEO. In a study of patients referred for ptosis surgery, 5.6% of cases had a neurogenic cause, and among these cases, the majority were due to serious underlying aetiologies (35.7% palsy of the 3^rd^ cranial nerve, 28.6% myasthenia gravis, 14.3% aberrant regeneration, 7.1% Horner’s syndrome). While myogenic causes (which broadly include conditions such as OPMD, CPEO, and myotonic dystrophy) were likewise uncommon in the study population (4.0% overall), 30% of the patients in this group had an underlying diagnosis of CPEO [24].

Horner’s syndrome, in its acquired form, is usually secondary to interruption of sympathetic innervation of the superior and inferior tarsal muscles due to trauma, certain tumours, or stroke. It is characterised not only by mild unilateral ptosis of the upper eyelid, but also the lower eyelid (i.e., slight elevation of the lower lid margin), ipsilateral pupillary miosis, facial anhidrosis, and a positive pupillary response (dilation) to topical phenylephrine (which can be used to differentiate between pre- and post-ganglionic Horner’s syndrome) or apraclonidine [5, 16, 39, 40]. Ptosis caused by 3^rd^ cranial nerve palsy—which innervates, among other muscles, the levator palpebrae superioris—has a unilateral and variable presentation but is typically accompanied by diplopia and a “down and out” position of the affected eye due to partial or complete muscular paresis [5, 16, 40]. Like with Horner’s syndrome, 3^rd^ cranial nerve palsy can be secondary to an acute event such as ischaemia, aneurysm, or trauma, or to compression of the nerve by an expanding mass. Because the 3^rd^ cranial nerve delivers most of the parasympathetic fibres destined for the eye, dilation of the ipsilateral pupil can be observed in some cases. Pupillary involvement requires neuroimaging for the presence of an aneurysm or of a tumour that may be compressing the nerve. Lack of pupillary involvement often suggests a microvascular cause, such as diabetes mellitus [5, 16, 40].

Ptosis can also be an early symptom of myasthenia gravis, a condition caused by autoantibody blocking or destruction of nicotinic acetylcholine receptors, and may be accompanied by external ophthalmoplegia [5, 16, 40]. It can present either unilaterally or bilaterally (symmetric or asymmetric), tends to worsen with fatigue, and can be identified in-office by a positive response (upper eyelid elevation) to the rest test or ice test [16, 40–42]. Furthermore, diagnosis can be confirmed via serologic testing for anti-acetylcholine receptor antibodies [16]. When myasthenia gravis is suspected, CT scanning is required, in order to identify potential thymic hyperplasia or thymoma [42]. Ptosis secondary to CPEO, a mitochondrial syndrome, can be accompanied by extraocular muscle weakness, particularly on upgaze, and reduced saccadic velocity, and requires evaluation for involvement of other systems [5, 15].

Evaluation of the periocular skin and soft tissues is essential to identifying or excluding ptosis secondary to a mass weighing down the upper eyelid [5, 16, 22]. Patients should be examined for suspicious lesions, such as basal cell or squamous cell carcinoma of the skin, or unusual masses beneath the skin. A lacrimal gland mass can present as upper eyelid ptosis, and potential aetiologies for lacrimal gland masses include lymphoma, adenoid cystic carcinoma, or pleomorphic adenoma, all of which require workup prior to considering ptosis as the diagnosis. Examination should include palpation of the superolateral portion of the upper eyelid beneath the tail of the brow near the orbital rim, and if a mass is suspected, referral to a specialist is recommended.

Also essential to the clinical workup is the exclusion of “pseudoptosis” conditions, which involve no pathology of the upper eyelid retractor muscles or levator aponeurosis, but instead are due to pathologies of other structures that indirectly affect eyelid position. Pseudoptosis can arise, for example, due to a range of mechanical (dermatochalasis, brow ptosis, floppy eyelid syndrome), anatomical (globe dystopias, globe asymmetry, ocular misalignment), or neurogenic (hemifacial spasm, blepharospasm) causes, or contralateral eyelid retraction (thyroid eye disease) [5, 16, 40]. The pathology specific to the various forms of pseudoptosis means that treatment targeting the upper eyelid muscles or aponeurosis is unlikely to resolve the condition, so when conducting the upper eyelid exam, it is important to identify any causes of pseudoptosis. Dermatochalasis, the presence of redundant upper eyelid skin, is identified by lifting the excess eyelid skin and performing an eyelid examination. If eyelid elevation and muscle function are normal, then ptosis is excluded [16]. Evaluation of the globe can identify dystopias such as enophthalmos, hyperglobus, hypoglobus, or asymmetry caused by phthisis bulbi, microphthalmia, or other conditions affecting globe size and giving the appearance of unilateral ptosis [16]. To differentiate ptosis from contralateral eyelid retraction due to thyroid eye disease, the ptotic eyelid can be lifted and the contralateral eye observed for relaxation indicative of compensatory retraction secondary to ptosis [16]. One may also assess whether there is lid lag on downward gaze, another indication of thyroid ophthalmopathy.

After appropriate examination of the ocular and periocular structures, assessment of upper eyelid function can be performed with a few simple measurements. The distance from the central pupillary light reflex to the central margin of the upper eyelid (marginal reflex distance 1 (MRD-1)) helps define the presence and severity of ptosis. In the normal eye, MRD-1 is typically 4–5 mm, and a decrease in this measure signals the presence of ptosis [5, 11, 16]. Less relevant in the context of acquired ptosis is MRD-2 (the distance from the centre of the pupillary light reflex to the lower eyelid margin with the eye in primary gaze) and MRD-3 (the distance from the pupillary light reflex to the upper eyelid margin with the eye in extreme upgaze). The MRD-3 measure is used to determine the degree of levator resection required in patients with congenital ptosis and vertical strabismus [43].

Eyelid crease height, the distance from the upper eyelid crease to the eyelid margin, can likewise be informative. Normal eyelid crease height generally ranges from 7 to 8 mm in males and 9–10 mm in females, and an increase in this measure can indicate disinsertion of the levator aponeurosis [44]. Palpebral fissure height is a measure of the distance between the upper and lower eyelid margins with the eye in primary gaze, with a normal value in the range of 10–12 mm. A decrease in palpebral fissure height can be an indicator of disinsertion of the levator aponeurosis from the tarsal plate [16, 44]. Levator function is more directly assessed using Berke’s method, in which frontalis muscle function is negated (by holding the brow) and the patient shifts from downgaze to upgaze. Levator function is classified based on the amount of upper eyelid excursion, from poor (0-4 mm lid elevation), to fair (5–11 mm), good (12–14 mm), and normal (>15 mm) [44]. Müller’s muscle function can be assessed using the phenylephrine test, in which a drop of the α-adrenergic agonist phenylephrine 2.5% is applied under the ptotic eyelid. A positive response to phenylephrine (eyelid elevation) is indicative of Müller’s muscle function and suggests that the patient is a candidate for Müller’s muscle-conjunctival resection [16, 44–48].

Visual field testing is an important tool for measuring any functional deficits caused by ptosis [7, 9, 11, 49]. The Goldmann Visual Field (GVF) Test is a manual kinetic perimetry test, in which the patient fixates on the centre of the testing field and indicates when they see moving illuminated targets of varying size and brightness in the peripheral field, and the visual field is mapped by the examiner [50]. The HVF Test is an automated static perimetry test using an HVF analyser, in which static illuminated targets briefly appear in the field and patients indicate when a target is seen. Most commonly, the HVF Test evaluates a 24° field (24-2 setting) using a 54-point grid [10, 50]. The LPFT is a modified HVF Test, specifically designed to assess superior visual field deficits caused by ptosis, that demonstrates high sensitivity, specificity, and positive/negative predictive value [10]. It is an automated, observer-independent, static perimetry test that evaluates a 48° range in the superior visual field, using a 4-row, 35-point grid. The centre of fixation on the LPFT is shifted 15° inferiorly to maximise testing of the superior field, enable more natural eyelid positioning, and prevent compensatory behaviours, such as brow elevation [10].

## Acquired ptosis treatment

The standard of care for ptosis management is surgical intervention. Elevation of the upper eyelid for functional or cosmetic purposes can be successfully achieved with a variety of techniques targeting the upper eyelid retractor muscles and aponeurosis [51–58], and the procedure (or combination of procedures) is selected based on underlying ptosis aetiology and severity (Table 4) [5, 44, 49]. Requisites for a functional indication include measurable decrease in eyelid elevation (typically defined as MRD-1 ≤ 2 mm) and accompanying superior visual field deficit, demonstrated via visual field testing [59]. Common procedures targeting Müller’s muscle include Müller’s muscle-conjunctival resection, in which Müller’s muscle and the overlying conjunctiva are excised using a posterior approach. This procedure is used for mild acquired ptosis or Horner’s syndrome with good levator function. Similarly, in the Fasanella-Servat procedure, the lower part of Müller’s muscle, overlying conjunctiva, and upper border of the tarsus are resected. This procedure is also typically reserved for mild acquired ptosis or Horner’s syndrome with good levator function, with the amount of muscle resected dependent on the degree of eyelid droop [5, 6, 44].

**Table 4:** Approaches to acquired ptosis treatment.

Surgical approaches	
Müller’s muscle	• Müller’s muscle-conjunctival resection or Fasanella-Servat procedure; used when ptosis is mild and levator function is good [5, 6, 44]
Levator or levator aponeurosis	• Levator advancement (aponeurosis repair); used when there is dehiscence or detachment of the levator aponeurosis and decreased levator function [5, 44] • Levator resection; amount of muscle resected increases with decreasing pre-surgical levator function; Whitnall’s ligament suspension used when levator function is poor [5, 44, 60]
Frontalis muscle	• Frontalis suspension; connection of the frontalis and upper eyelid using a sling when levator function is poor and frontalis function is good [5, 44]
Non-surgical approaches	
Observation/‘watch and wait’	• Conservative approach for transient ptosis (self-resolving) or slowly progressing acquired ptosis (requiring intervention when more severe)
Mechanical intervention (eye crutches, adhesives)	• Temporary solutions with limited benefit and concerns related to comfort, convenience
Scleral contact lenses	• Mechanical intervention, by which upper eyelid elevation is increased during lens wear [63–65] • Applications reported in case series limited to complex ptosis [63, 65]
Off-label topical α-adrenergic agents	• Ophthalmic solutions (phenylephrine, apraclonidine, brimonidine, naphazoline) used for other applications, but with effects on Müller’s muscle • Studies limited in scope demonstrate some effects on upper eyelid elevation after administration [37, 38, 66–68, 72–75], but also ocular and non-ocular side effects associated with short term or long-term (in other ophthalmic applications) use [45, 69, 71–73]
Topical oxymetazoline	• Approved α-adrenergic agent (0.1% ophthalmic solution) administered once daily; exerts effects at Müller’s muscle • Demonstrated significant improvement of superior visual field deficits and upper eyelid elevation, and limited ocular adverse events, over 42 days of treatment in phase 3 trials [81]

If there is dehiscence or disinsertion of the aponeurosis but levator muscle function remains good, levator muscle advancement (aponeurosis repair), using an anterior or posterior approach, can be performed. Levator resection is used in cases in which levator function is in the fair-to-good range (> 4 mm), with the amount of muscle resected dependent upon the degree of pre-surgical levator function [5, 44]. If levator function is poor, the desired upper eyelid elevation can be provided via Whitnall’s ligament suspension, in which aponeurotic resection is followed by suturing of Whitnall’s ligament to the tarsal plate and suspension of the ligament to the periosteum of the superior orbital rim [44, 60]. If levator function is poor and frontalis function is good, as is the case in many patients with congenital ptosis, a subcutaneous sling to connect the frontalis muscle to the upper eyelid can also be used. This procedure can also be used for acquired ptosis with a myogenic or neurogenic cause [5, 44]. For patients with both ptosis and dermatochalasis, a combination of ptosis repair and upper lid blepharoplasty procedures may be appropriate [16].

Surgical intervention has been demonstrated to improve elevation of the upper eyelid and superior visual field deficits, and these clinical improvements can accordingly improve patients’ performance of activities of daily living and HRQoL outcomes. As noted in Acquired ptosis overview, prevalence, and impacts above, patients who undergo ptosis surgery report improved ability to perform common visual tasks and activities of daily living, leading to an improved functional index [12, 13, 49]. Despite these well-established benefits of surgery, however, it is not an ideal approach for some patients. In many cases, ptosis is not severe enough, with respect to appearance or functional deficit, in the view of the surgeon, patient, or payer to warrant surgical intervention. Furthermore, the potential benefits of surgical intervention must be weighed against risks of unwanted side effects or outcomes. The most common risks associated with ptosis surgery range from temporary adverse events (AEs) such as bleeding, bruising, and infection, to more persistent AEs such as scarring, eyelid crease abnormalities, over- or under-correction, and eyelid asymmetry. There are also secondary risks to over-correction, including lagophthalmos and exposure keratopathy [5]. Unilateral ptosis in particular can present unique challenges with respect to achieving desired symmetry. The levator muscles are yoke muscles bilaterally innervated by the same afferent input, which increases when one or both eyes is ptotic. In the case of unilateral ptosis, afferent input to the levator muscle of both eyes increases, resulting in elevation of the ptotic eyelid, but also the contralateral eyelid (pseudoretraction). Following unilateral ptosis surgery, the compensatory decrease in afferent input to the formerly ptotic eyelid is paralleled by a decrease in input to the contralateral eye, causing it to droop and result in secondary contralateral ptosis (Hering’s phenomenon), and the potential need for revision surgery [6, 61]. A thorough examination for pseudoretraction is therefore essential in cases of unilateral ptosis that are candidates for surgery.

A retrospective analysis of 1519 patients who underwent ptosis surgery revealed that revision was required in 8.7% of cases, with a 6.8% revision rate in patients who underwent a posterior-approach procedure and a 9.5% revision rate in those who underwent an anterior-approach procedure [62]. Over- and under-correction were identified as the predominant reasons for revision, and the mean time to revision was 24.6 ± 25.2 weeks [62]. Among subjects who underwent unilateral ptosis surgery (355 total), 5.1% had a postoperative contralateral ptosis that prompted revision surgery [62].

Non-surgical approaches to managing ptosis—and clinical evidence supporting any of these approaches—have been comparatively limited (Table 4). The most conservative approaches include simple observation and (in the case of transient ptosis) waiting for self-resolution, as well as the use of mechanical interventions such as eye crutches and adhesives [5]. These mechanical interventions are, at best, temporary solutions that may present more inconvenience to patients than benefit. Reports have also described the use of scleral contact lenses for the treatment of complex ptosis [63–65]. While preferable to eye crutches or adhesives given the opportunity for better comfort and cosmesis, the principle of scleral contact lens use is similar, with the lens providing mechanical support to raise the ptotic upper eyelid. A retrospective analysis of scleral lens wear indications at Moorfields Eye Hospital in the United Kingdom revealed ptosis as the indication for 1.7% of eyes evaluated [64]. A case review of 10 patients with complex ptosis who were scleral contact lens wearers revealed objective clinical improvements in mean palpebral aperture and MRD-1 during lens wear, but when patients subjectively assessed cosmesis, the effect of the lenses was judged as “moderate” or “poor” for 78% of eyes assessed [65]. A subsequent study of three patients with complex ptosis similarly found objective increases in palpebral aperture and MRD-1, and scleral lens wear was reported by patients to be comfortable [63]. Still, the overall evidence for scleral lens wear is limited, as are its applications in clinical practice, at least in part because contact lens wear itself can be associated with the development of aponeurotic ptosis [25–29].

Topical sympathomimetic agents, including phenylephrine, apraclonidine, brimonidine, and naphazoline, have ophthalmic applications outside of ptosis, but have also been evaluated for their effects on the ptotic upper eyelid based on their potential to activate Müller’s muscle, which has been shown to express the α_1_/α_1D_, α_2A_, and α_2C_ adrenergic receptor subtypes [17–19]. A retrospective study of patients with dehiscence of the levator aponeurosis found that 78% of eyes instilled with a single drop of 10% phenylephrine showed a positive response (i.e., an increase in MRD-1), and that responsiveness did not depend on ptosis severity or levator function. There was an association between responsiveness and ptosis aetiology, however, with a 77% of eyes with ptosis caused by previous ocular surgery showing a ≥1.5 mm increase in MRD-1, versus 42% of eyes with aging as the only identifiable cause showing the same degree of increase [66]. While these effects on the upper eyelid are intriguing, clinically significant pupil dilation is also observed in the majority of patients following phenylephrine instillation [45], limiting its utility for ptosis treatment.

Apraclonidine demonstrates strong agonist activity at α_2_-adrenergic receptors and weaker agonist activity at α_1_-adrenergic receptors. Via its α_1_-adrenergic receptor-mediated effects, topical apraclonidine can temporarily reverse anisocoria due to Horner’s syndrome, thus supporting this diagnosis [16]. A case report also revealed elevation of the upper eyelid in three patients with Horner’s syndrome after instillation of 0.5% apraclonidine [67]. A larger evaluation of the effect of apraclonidine on upper eyelid elevation was conducted in 100 non-ptotic subjects, demonstrating small mean increases in MRD-1 at 30 and 45 min post-instillation that were hypothesised to be a result of stimulation of postsynaptic α_1_-adrenergic receptors [68].

Apraclonidine’s effect in ptotic patients has also been evaluated in a number of small-scale studies. A retrospective case series examining 7 patients with ptosis following cosmetic botulinum toxin injection revealed a mild effect of apraclonidine 0.5% on ptosis, but only when used within 4–6 weeks of ptosis self-resolution, suggesting that apraclonidine response might help predict resolution time [37]. A later case series evaluated the effect of administering two drops of apraclonidine 0.5% in a cohort of 6 patients with ptosis resulting from botulinum toxin injection and another with Horner’s syndrome, showing improvement in upper eyelid elevation 20–30 min following instillation [38]. A prospective study enroling 26 patients scheduled for ptosis surgery revealed variability in upper eyelid responsiveness to apraclonidine 0.5%, and immunohistochemical examination of resected Müller’s muscle tissue revealed higher expression of the α_1D_-adrenergic receptor subtype in responsive eyelids, suggesting that the drug’s effect on the upper eyelid is mediated at least in part by agonism of α_1D_ receptors [18].

While studies of limited apraclonidine dosing in patients with ptosis have revealed no notable safety concerns, prospective studies of its chronic use for other ophthalmic applications (glaucoma, ocular hypertension) have reported ocular (including decreased visual acuity and allergic conjunctivitis) and non-ocular (such as dry mouth and contact dermatitis) side effects that have led to discontinuation of use [69–71]. In the context of treating ptosis, this side effect profile is likely undesirable to patients and practitioners.

Other adrenergic agents have also been evaluated for potential applications to ptosis. A study of 20 healthy adult volunteers found significant elevation of the upper eyelid for up to 2 h following instillation of naphazoline 0.05% (a preferential α_2_-adrenergic receptor agonist), but not brimonidine 0.2% or phenylephrine 0.12% [72]. Topical naphazoline was also reported to improve upper eyelid elevation in a cohort of 12 patients with myopathic ptosis, with limited ocular side effects, but tachyphylaxis was observed with frequent daily dosing over a period of weeks [73]. Naphazoline use has also been reported in ptotic patients with myasthenia gravis, with topical use providing observable opening of the eye in 70% of enroled patients [74]. A case study in a single patient with anterior laminectomy-induced Horner’s syndrome revealed a positive effect of twice-daily unilateral administration of brimonidine tartrate 0.1% for 3 months [75]. While suggestive of possible applications in treating some cases of ptosis, the data regarding the adrenergic agents phenylephrine, apraclonidine, brimonidine, and naphazoline are limited in scope and none of these agents are approved for the treatment of ptosis. An important consideration in the context of treating ptosis is that chronic use of some α-adrenergic agents for applications such as lowering of intraocular pressure in patients with glaucoma, can be associated with tachyphylaxis, and that this may depend on the agent’s α-adrenergic subtype selectivity [69, 76–78].

More recently, the efficacy and safety of an oxymetazoline 0.1% ophthalmic solution approved for the treatment of acquired ptosis (Upneeq^TM^, RVL Pharmaceuticals, Inc., Bridgewater, NJ, USA) has been reported. Oxymetazoline is a direct-acting α_1_- and α_2_-adrenergic receptor agonist [79, 80]. Like other α-adrenergic agents, oxymetazoline is thought to act by stimulating contraction of Müller’s muscle. Evidence from two phase 3 clinical trials revealed that once-daily use of oxymetazoline 0.1% for 42 days significantly improved the superior visual field and upper eyelid elevation in patients with acquired ptosis and accompanying superior visual field deficit [81]. Using the LPFT, these studies demonstrated mean 5.9 ± 6.4 and 7.1 ± 5.9 point improvements in the superior visual field on treatment days 1 (6 h post-instillation) and 14 (2 h post-instillation), respectively, both of which were statistically superior to the mean change observed with vehicle at the corresponding time points (day 1: 1.8 ± 4.1 points; day 14: 2.4 ± 5.5 points). Similarly, MRD-1 measurements showed 0.96 ± 0.89 mm and 1.16 ± 0.87 mm improvements with oxymetazoline 0.1% on treatment days 1 and 14, respectively, in comparison to 0.50 ± 0.81 mm and 0.50 ± 0.80 mm with vehicle at the same time points [81]. Importantly, these studies showed that oxymetazoline 0.1% was effective after administration of a single drop beginning on treatment day 1 (measured 6 h post-instillation) and was associated with relatively low AE rates, making it a particularly intriguing non-invasive treatment option for acquired ptosis [81]. No tachyphylaxis was reported over 42 days of once-daily use in these studies of oxymetazoline 0.1%, however longer-duration evaluation is required to more thoroughly explore the potential effects of chronic use.

## Summary, conclusions, and future directions

The prevalence and wide-ranging clinical and functional implications of acquired ptosis make timely and accurate diagnosis and treatment important for eye care practitioners. Acquired ptosis is most often due to age-related changes in the upper eyelid retractor muscles [24, 25], but underlying causes are varied, and many practices and interventions common in eye care today, such as contact lens wear and cataract and glaucoma procedures, can in fact contribute to the development of transient or more persistent forms of ptosis [25–35]. Along with other aetiologies discussed in this article, all warrant full examination and evaluation of treatment opportunities.

Surgery is an effective treatment option for ptosis, but non-surgical approaches have been extremely limited in both number and effectiveness. Because treatment via surgical intervention may be limited to a relatively small proportion of patients, finding ways to incorporate novel non-surgical therapeutic options into practice presents the potential to treat a wider range of patients. The evidence regarding a newly approved pharmacologic agent for the treatment of acquired ptosis [81] is therefore encouraging and suggests the opportunity to offer effective non-surgical treatment. For eye care practitioners—and indeed a range of health care professionals—the availability of an approved pharmacological option might help shift from a “detection and referral” approach to a “diagnosis and treatment” approach, with referral for surgery when appropriate. Furthermore, the expansion of therapeutic options may help to improve the patient focus of treatment, by allowing for the use of surgical and non-surgical approaches as appropriate based on underlying ptosis aetiology, severity, and patient preference.

While advances in ptosis treatment are encouraging, these remain only part of the clinical equation. To effectively treat ptosis, timely and accurate diagnosis is essential. In particular, comprehensive clinical examination and differential diagnosis are critical to understanding whether a patient’s ptosis is due to primary pathology of the upper eyelid retractor muscles—and can thus be effectively managed by surgical or pharmacological means targeting the upper eyelid—or whether the underlying cause is a more serious underlying neurological condition requiring different intervention. While in many cases ptosis might only be evaluated and treated when its onset is sudden or severity is high, examination of the upper eyelid for mild-to-moderate or progressive cases can be incorporated into the comprehensive eye exam with relative ease. Together with a focus on awareness and diagnosis, focused surgical or non-surgical treatment based on the clinical evidence offers the promise of improved ptosis treatment for more patients.
